# Burnout and Health Issues among Prehospital Personnel in Taiwan Fire Departments during a Sudden Spike in Community COVID-19 Cases: A Cross-Sectional Study

**DOI:** 10.3390/ijerph19042257

**Published:** 2022-02-16

**Authors:** Yu-Tung Chang, Yih-Jin Hu

**Affiliations:** Department of Health Promotion and Health Education, National Taiwan Normal University, Taipei City 106, Taiwan; emtpjack@gmail.com

**Keywords:** COVID-19, emergency worker workload, emergency worker burnout, pressure, emergency medical services

## Abstract

A spike in COVID-19 cases in Taiwan’s communities caused a significant increase in workload and infection concerns among prehospital personnel working in Taiwan fire departments. The present study was aimed at investigating their health status during this period. The target population was prehospital personnel who are from Taiwan fire departments, and who responded to COVID-19 patients during the community outbreak period. A questionnaire was employed to assess their physical and mental health status. The results showed that prehospital personnel suffered from moderate to severe degrees of burnout. Workload, body burden, and perceived pressure increased significantly during this period. Participants received more support from friends, family, and colleagues than they did from authorities. The paramedics reported higher scores for personal burnout than the emergency medical technicians (EMTs). Compared to non-COVID-19 response units, special COVID-19 response units reported higher scores for workload, body burden, and supportive environment. The results suggested that personal and work-related burnout were associated with higher perceived pressure. This study is the first investigation of physical and mental health burdens among prehospital personnel in Taiwan fire departments during the COVID-19 pandemic. The physical and mental health status of these personnel should be continuously monitored, and intervention provided as necessary.

## 1. Introduction

The COVID-19 pandemic has been impacting societies around the world since late 2019. In Taiwan, a spike in the number of COVID-19 cases caused a significant increase in the workload of hospital and prehospital healthcare providers in communities between May and September in 2021 [1]. During this period, health and emergency authorities assigned emergency ambulance crews in fire departments to transport patients with unstable COVID-19 conditions to hospitals, whereas minor or asymptomatic cases were transported to quarantine facilities. Emergency ambulance crews comprising emergency medical technicians (EMTs) and paramedics working in fire departments were required to wear full personal protective equipment, and drive ambulances for hours daily to transport COVID-19 patients to minimize the spread of COVID-19 in the community [1]. Some EMTs and paramedics reported suffering from heat-related illness and psychological stress after operating COVID-19 patient transports [1].

Studies have shown that dramatic changes in the work environment, and elevated risk levels in hospital and prehospital settings have induced significant pressure, anxiety, depression, and burnout in healthcare workers during the COVID-19 pandemic [2,3,4,5,6,7,8,9,10]. One study also suggested that building a supportive environment and resilience capability may be beneficial to alleviating stress, anxiety, and pressure during this period [11]. In recent studies, measurements of mental health status were mainly focused on healthcare workers in hospitals. However, health impacts to EMTs and paramedics, who work in environments that pose a high risk of infection and uncertainty, have been underestimated, and require intervention and greater attention from society and relevant authorities [11,12].

Thus, the present study aimed to measure factors related to physical and mental health among EMTs and paramedics in Taiwan fire departments, and investigate the association among these factors during the spike in the number of COVID-19 cases in Taiwanese communities.

## 2. Materials and Methods

This cross-sectional study was conducted between 2 September and 1 October 2021. The target population was EMTs and paramedics from Taiwan fire departments who responded to transport or care of any patients who tested positive for COVID-19 during a community outbreak since May 2021. Authorities assigned groups of experienced EMTs and paramedics to be responsible for the transportation of patients with COVID-19 in different jurisdictions. At the time of this study, approximately 10,000 frontline EMTs and paramedics were working in Taiwan fire departments [13]. Based on COVID-19 response reports from various fire departments, the estimated size of the target population in this study was 500 (5%) EMTs and paramedics [1].

This study adopted a structured questionnaire to assess the physical and mental health status of EMTs and paramedics. The questionnaire was constructed using Google Forms, and distributed via email and social media communication networks between EMTs and paramedics in different jurisdictions. The present study was reviewed and approved by the Ethical Committee at En Chu Kong Hospital, Taiwan (ECKIRB1100803).

The questionnaire comprised different aspects related to physical and mental health, including 6 sections: demographics and health behavior (smoking, alcohol consumption, and exercise), personal and work-related burnout, workload and body burden, perceived pressure, supportive environment, and expression of thoughts and feelings in this period. This questionnaire was evaluated by experts validity and pre-test that obtained the reliability of Cronbach’s alpha 0.9 and KMO value 0.8.

In completing the questionnaire, the participants retrospectively evaluated their physical and mental status during the community outbreak. The contents in the burnout section were adopted from the Taiwan Ministry of Labor burnout measurement tool, with 6 questions used to measure personal-related burnout, and 7 questions to measure work-related burnout. Personal-related burnout results were interpreted as severe for scores higher than 70, moderate for scores between 50 and 70, and minor for scores below 50; work-related burnout results were interpreted as severe for scores higher than 60, moderate for scores between 45 and 60, and minor for scores below 45 [14].

The other sections exploring mental health-related status included workload and body burden, perceived pressure, and supportive environment. These were measured using a 5-point Likert scale (1 = significant increase, 2 = increase, 3 = no difference, 4 = decrease, 5 = significant decrease). Scores for these sections were re-calculated into a scale from 0 to 100. The average scores in each section were taken to reflect the degree of mental status among the participants. The final section was an open-ended question for participants to express their thoughts, experiences, and feelings during the spike in community cases.

Continuous variables were described using the mean and standard deviation (SD). Categorical variables were presented as frequencies and percentages. The mean comparison of continuous variables between groups was computed with Student’s *t* test and an analysis of variance (ANOVA). Correlations between continuous variables were analyzed using Pearson’s coefficient and the chi-squared test. Linear regression was conducted to investigate the effects of demographics, health behavior, and mental health-related status on personal and work-related burnout scores. Path analysis was also conducted to demonstrate the degree of association between the variables. For all results, *p* < 0.05 was considered statistically significant. Analyses were performed using SPSS 23 and LISREL 8.8 statistical software.

## 3. Results

### 3.1. Participant Characteristics

Table 1 summarized the demographic data of the participants. The sample comprised 187 participants from Taiwan fire departments; 173 (92.5%) were male, and 14 (7.5%) were female. Their mean age was 35.4 (±6.5) years, and average work experience was 11.8 (±6.8) years. The average number of dependents among the participants was 2.1 (±1.3). There were 118 (63.1%) participants who were married, 66 (35.3%) who were unmarried, and 3 (1.6%) who were divorced. Regarding education level, 76 (40.6%) held an undergraduate degree, 44 (23.5%) had completed a postgraduate degree, and 67 (35.8%) held a diploma degree. Of all participants, 112 (59.9%) were paramedics, and 75 (40.1%) were EMTs.

Regarding the participants’ jurisdiction, 93 (49.8%) of the participants were from COVID-19 response units, and 94 (50.2%) were from non-COVID-19 response units. Of all EMTs and paramedics in this study, 73 (39%) were from New Taipei City, 31 (16.6%) were from Taichung City, 21 (11.2%) were from Kaohsiung City, 13 (7%) were from Hsinchu City, 12 (6.4%) were from Tainan City, 11 (5.9%) were from Taipei City, 8 (4.3%) were from Taoyuan City, 6 (3.2%) were from Hsinchu County, and 12 (6.4%) were from other jurisdictions in Taiwan. Regarding financial status, 9 (4.8%) participants reported living below the poverty line, 151 (80.7%) were middle class, and 27 (14.4%) were in the upper class. Regarding religious beliefs, 101 (54%) were without a specific religion, 25 (13.4%) were Buddhist, 48 (25.7%) were Taoist, 8 (4.3%) were Christian, and 5 (2.7%) followed other religions.

This study investigated health-related behaviors, including smoking, drinking, and exercise. The results showed that 156 (83.4%) were non-smokers, 14 (7.5%) smoked <1 pack of cigarettes per week, 12 (6.4%) smoked 1–2 packs per week, and 5 (2.7%) smoked >1 pack per day. Regarding their alcohol consumption habits, 63 (33.7%) of the participants reported no habit, 113 (60.4%) drink alcohol occasionally, and 11 (5.9%) drink more than 3 times per week. For exercise habits, 36 (19.3%) of the participants reported that they had no exercise habit, 100 (53.5%) exercised 1–2 times per week, and 51 (27.3%) of them exercised more than 3 times per week.

The average scores obtained from the questions in each section were used to reflect the status of each variable related to mental health. Table 2 lists the mean and SD for each variable. The average personal burnout score was 55.9 (±22.4), and for work-related burnout, was 51.3 (±22.0). The burnout scores indicated that EMTs and paramedics from Taiwan fire departments were suffering at least a moderate level of burnout during the investigation period. Workload, body burden, and perceived pressure scores were 83.1 (±11.2), 64.7 (±6.5), and 72.6 (±13.4), respectively, including a moderate-to-significant increase in these areas during the study period. The scores for supportive environment reflected a moderate increase during this period.

### 3.2. Characteristics and Mental Health-Related Status

The scores for mental health status differed between the characteristics (Table 3). Paramedics revealed higher personal burnout scores than those at the EMT-2 level (58.5 vs. 52.0, *p* < 0.05). Special COVID-19 response units showed higher scores in workload (84.4 vs. 82.6, *p* < 0.05), body burden (66.2 vs. 63.8, *p* < 0.05), and supportive environment (70.9 vs. 69.5, *p* < 0.05) than non-COVID-19 response units. Participants whose jurisdiction was in New Taipei City showed a significantly greater workload (85.9 vs. 78.3, *p* < 0.05) and supportive environment (72.9 vs. 65.2, *p* < 0.05) than those in Taichung City. Participants below the poverty line reported higher scores for work-related burnout (67.4 vs. 44.9, *p* < 0.05) and perceived pressure (81.2 vs. 68.0, *p* < 0.05) than those who were upper class. Body burden and perceived pressure scores among those who reported consuming alcohol occasionally were higher than those who did not habitually consume alcohol (65.6 vs. 62.8). Participants who exercised 1–2 times per week had significantly higher scores in personal-related burnout (61.2 vs. 47.3, *p* < 0.05), work-related burnout (56.5 vs. 42.7, *p* < 0.05), and perceived pressure (75.6 vs. 68.7, *p* < 0.05) in comparison to those who exercised 3 times per week.

### 3.3. Association of Burnout and Observed Factors

Two linear regressions were calculated to predict personal and work-related burnout based on the demographic variables and scores for workload, body burden, perceived pressure, and supportive environment (Table 4). For predicting the personal-related burnout score, a significant regression equation was found ((F_(13, 173)_ = 70.31, *p* < 0.001); R^2^ = 0.841). Participants’ predicted personal-related burnout was equal to −32.35 to 0.42 (work experience) + 0.19 (workload) + 0.83 (work-related burnout). Participants’ personal-related burnout decreased by 0.42 for each year of work experience, and increased by 0.19 and 0.83 for each degree of workload and work-related burnout. In the prediction for work-related burnout, a significant regression equation was found (F_(22, 164)_ = 43.07, *p* < 0.001); R^2^ = 0.852). Participants’ predicted personal-related burnout was equal to 10.36 + 0.25 (perceived pressure) + 0.77 (personal-related burnout). Participants’ work-related burnout increased by 0.25 and 0.77 for each degree of pressure and personal-related burnout.

**Figure 1:**
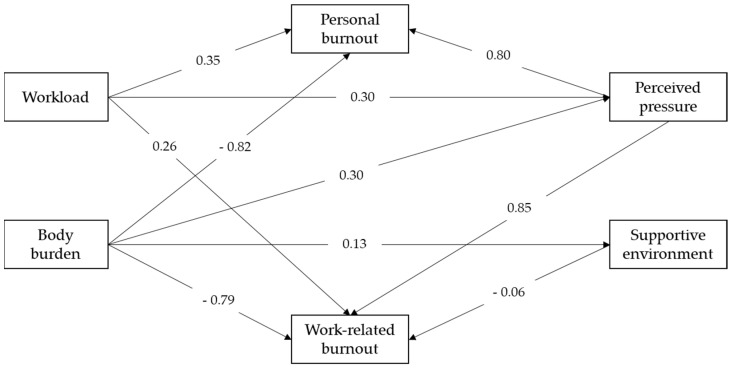
Path analysis diagram and standardized coefficients of burnout and work-related health issues of prehospital personnel.

Figure 1 displays the results of the initial path analysis model of work-related variables affecting personal and work-related burnout. The goodness-of-fit resulted a chi-squared of 4.145 (*p* = 0.387) with degrees of freedom of 4. The goodness-of-fit index (GFI) was 0.993, and the adjusted GFI was 0.963. Fit indices, such as comparative fit index (CFI), normal fit index (NFI), and relative fit index (RFI), were all over 0.9 in acceptance level. This path analysis showed that personal burnout was directly affected by workload (0.35), body burden (−0.82), and perceived pressure (0.80). Work-related burnout was directly affected by workload (0.26), perceived pressure (0.85), body burden (−0.79), and supportive environment (−0.06). From the results above, perceived pressure affected both personal-related and work-related burnout compared to other work-related variables. Body burden produced negative effects to both personal-related and work-related burnout. The effect of body burden to personal burnout was also mediated by perceived pressure. The effect of body burden to work-related burnout was mediated by a supportive environment.

### 3.4. Qualitative Feedback from Participants

There were 96 participants who responded to the open-ended question in the questionnaire. By excluding emotional expressions, there were 40 responses that identified specific issues during this sudden spike in community COVID-19 cases. A lack of personal protective equipment decontamination supply occupied 22 responses. There were 15 responses that highlighted the situation of lack of rest time during this period. There were three responses that described the situation of perceived pressure from supervisors or authorities.

## 4. Discussion

Only a few studies have investigated the mental health condition of EMTs and paramedics, which may contribute to the worsening physical and mental condition among prehospital healthcare workers being underestimated [1,11,12]. The present study assessed EMTs and paramedics in terms of perceived pressure, workload, physical burden, and burnout status compared to these conditions before the recent COVID-19 community outbreak in Taiwan. The increase in workload and the physical burden was found to be associated with an increase in burnout and perceived pressure among EMTs and paramedics in Taiwan. These results may provide a warning for EMTs and paramedics, in that this could also further compromise their well-being, as well as the safety of patients and the quality of care given to the patients during this period. As this pandemic lasted for more than 2 years, EMTs and paramedics have been exposed to a certain degree of pressure, burnout, fatigue, and other physical and mental health conditions. This situation may become a public health concern that needs to be addressed by relevant authorities.

Healthcare providers around the world have been experiencing various levels of perceived pressure, anxiety, depression, burnout, and post-traumatic stress disorder (PTSD) during the COVID-19 outbreak since late 2019 [2,3,4,5,6,7,8,9,10,15,16,17,18,19]. Although the situation in Taiwan has not been as severe as in other countries, the present study still detected an increase in perceived pressure, physical and mental burden, and burnout among Taiwan prehospital healthcare providers. The total case load and the length of the outbreak period may be associated with the level of mental stress among health care workers [4,18]. The peak of the outbreak in communities in Taiwan was almost 5 months in length, and this might not allow for a clear observation of the negative effects experienced by EMTs and paramedics, at least not to the extent that it can be confirmed that their health has been significantly compromised, or that they have developed PTSD. However, the results of this study still suggest that relevant authorities in Taiwan should commence monitoring the physical and mental health status of EMTs and paramedics, and prepare long-term prevention and intervention programs for them.

Throughout this pandemic, health-care workers have been working in an environment that is high in risk, uncertainty, and stress [2,3,8,15]. Many studies have suggested that it is important to establish a supportive work environment for healthcare workers to relieve pressure and stress [5,10,15,16,18]. In the present investigation, family, friends, and colleagues were the main sources of support for EMTs and paramedics during the community outbreak, which previous studies have also indicated. However, the results indicated the opposite concerning the support EMTs and paramedics have received from relevant authorities. From the qualitative results, the EMTs and paramedics reported that their pressure and stress were from authorities, primarily due to a lack of PPE, and the authorities not considering their fatigue levels in their response planning. Many responses from the participants also highlighted that EMTs and paramedics are not recognized as health-care professionals in Taiwan, resulting in resources not being fairly distributed compared to other health care professionals during this public health crisis. This became a trust issue for both prehospital care providers and their authorities. The authorities should thus consider including EMTs and paramedics as a part of the healthcare system, instead of classifying them only as emergency and rescue responders.

The open-ended question in the questionnaire was aimed at allowing the participants to express more about their thoughts and feelings during the community outbreak. Most of the responses expressed negative thoughts and feelings about the outbreak, including their fatigue and fears, and the misleading message from their authorities to the media. EMTs and paramedics work in an uncertain environment that brings them into contact with potential COVID-19 cases without early notification. Feelings of pressure, stress, anxiety and even anger from EMTs and paramedics in Taiwan were foreseeable under this circumstance. Based on this atmosphere among prehospital care providers, the participants also reported that they have been re-evaluating their roles and the meaning of being an EMT and paramedic in Taiwan’s fire departments. In addition, when the community infection became under control, the authorities started to require faster ambulance response times, which shortened the time responders had to properly equip PPE, and may compromise personal health, and be in breach of relevant safety policies. The control and prevention of EMTs and paramedics being infected during the COVID-19 pandemic should always be the primary consideration compared to other daily routines key performance indicators (KPIs), such as 90 s for gear-up and drive-out, as these standards were implemented before the outbreak [15,18]. Also, being assigned a lower priority for being vaccinated and receiving relevant compensation could place severe strain on the trust between prehospital care providers and their authorities.

A limitation of this study was that it focused on a narrow sample. The study can be considered a pilot study for investigating the current physical and mental condition of EMTs and paramedics working with fire departments in Taiwan. Emergency medical services also include private ambulance services, prehospital nurses, as well as physicians and nurses in emergency departments. In the future, target samples following this line of research should include all frontline emergency healthcare workers to build up a comprehensive understanding of how their physical and mental health status has been impacted by COVID-19.

## 5. Conclusions

This study is the first investigation of the physical and mental health burden of EMTs and paramedics in Taiwan’s fire departments during the COVID-19 pandemic. The results revealed an increase in perceived pressure, burnout, and health burden among those who have been responding to highly contagious patients in the community. Society and the authorities must provide continuous support to prehospital care providers who are risking their lives to serve the community during this pandemic. Their physical and mental health status should be continuously monitored, and interventions should be provided where necessary.

## Figures and Tables

**Table 1:** Demographic data of the participants, shown as frequencies and percentages. (*n* = 187).

Characteristics	Frequency (%)	Mean ± SD
Age		35.4 ± 6.5
Working experience (years)		11.8 ± 6.8
Number of dependents		2.1 ± 1.3
Gender		
Male	173 (92.5)	
Female	14 (7.5)	
Marriage		
Not married	66 (35.3)	
Married	118 (63.1)	
Divorced	3 (1.6)	
Education		
Diploma	67 (35.8)	
Undergraduate	76 (40.6)	
Postgraduate	44 (23.5)	
EMTs Level		
EMTs-2	112 (59.9)	
Paramedic	75 (40.1)	
Service Unit		
COVID-19 response units	93 (49.8)	
Non-COVID-19 response units	94 (50.2)	
Jurisdictions		
New Taipei City	73 (39.0)	
Taichung City	31 (16.6)	
Kaohsiung City	21 (11.2)	
Hsinchu City	13 (7.0)	
Tainan City	12 (6.4)	
Taipei City	11 (5.9)	
Taoyuan City	8 (4.3)	
Hsinchu County	6 (3.2)	
Others	12 (6.4)	
Financial status		
Below the poverty line	9 (4.8)	
Middle class	151 (80.7)	
Upper class	27 (14.4)	
Religion		
No religion	101 (54.0)	
Buddhist	25 (13.4)	
Taoist	48 (25.7)	
Christian	8 (4.3)	
Others	5 (2.7)	
Smoking habit		…
Non-smoker	156 (83.4)	
Less than 1 pack per week	14 (7.5)	
1–2 packs per week	12 (6.4)	
More than 1 pack per day	5 (2.7)	
Alcohol consumption habit		…
No drinking habit	63 (33.7)	
Drink occasionally	113 (60.4)	
Drink more than 3 times per week	11 (5.9)	
Exercise habit		
No exercise habit	36 (19.3)	
1–2 times per week	100 (53.5)	
More than 3 times per week	51 (27.3)	

**Table 2:** Mental health-related variables, given as the mean and SD.

Variables	Mean ± SD
Personal-related burnout	55.9 ± 22.4
Work-related burnout	51.3 ± 22.0
Workload	83.1 ± 11.2
Body burden	64.7 ± 6.5
Perceived pressure	72.6 ± 13.4
Supportive environment	69.7 ± 11.5

**Table 3:** Comparison of mental health-related scores within characteristic groups.

Variables	*n*	Workload	*p*	Body Burden	*p*	Personal Burnout	*p*	Work Burnout	*p*	Perceived Pressure	*p*	Supportive Environment	*p*
Gender													
Male	173	83.17 ± 11.4	0.742	64.8 ± 6.6	0.617	56.6 ± 21.8	0.149	51.9 ± 21.5	0.189	72.9 ± 13.5	0.383	70.0 ± 11.4	0.249
Female	14	82.1 ± 9.5		63.8 ± 6.2		47.6 ± 28.1		43.8 ± 26.7		69.6 ± 11.6		66.3 ± 11.8	
Marriage													
Not married	66	84.4 ± 9.3	0.456	64.1 ± 4.8	0.619	53.3 ± 22.3	0.303	48.5 ± 21.3	0.096	71.0 ± 10.8	0.088	67.3 ± 11.8	0.088
Married	118	82.4 ± 12.2		65.1 ± 7.4		57.6 ± 22.3		53.3 ± 22.0		73.9 ± 14.4		70.9 ± 11.2	
Divorced	3	80.0 ± 8.8		64.8 ± 5.5		44.4 ± 20.9		30.9 ± 26.8		59.6 ± 14.4		74.8 ± 5.1	
Education													
Diploma	67	85.3 ± 9.8	0.093	64.5 ± 6.0	0.136	56.5 ± 19.4	0.608	52.8 ± 17.5	0.398	73.2 ± 10.9	0.335	69.9 ± 9.7	0.366
Undergraduate	76	81.2 ± 12.7		65.7 ± 6.8		57.1 ± 22.8		52.2 ± 23.6		73.6 ± 14.7		68.5 ± 11.7	
Postgraduate	44	82.8 ± 10.3		63.3 ± 6.7		53.0 ± 25.8		47.4 ± 25.0		70.0 ± 14.4		71.6 ± 13.3	
EMTs level													
(1) EMTs-2	112	83.8 ± 11.4	0.257	64.8 ± 6.9	0.846	58.5 ± 22.9	0.048 * (1) > (2)	53.3 ± 21.5	0.118	74.2 ± 13.0	0.055	70.1 ± 10.8	0.572
(2) Paramedic	75	81.9 ± 11.0		64.6 ± 6.0		52.0 ± 21.1		48.2 ± 22.3		70.3 ± 13.6		69.1 ± 12.4	
Service units													
(1) COVID-19 Units	93	84.4 ± 10.1	0.008 * (1) > (2)	66.2 ± 6.45	0.002 * (1) > (2)	56.6 ± 21.0	0.113	51.4 ± 21.4	0.299	72.4 ± 13.1	0.112	70.9 ± 11.7	0.017 * (1) > (2)
(2) Non- COVID-19 Units	94	82.6 ± 10.5		63.8 ± 9.0		56.7 ± 22.4		52.4 ± 21.7		73.9 ± 13.1		69.5 ± 10.3	
Jurisdictions													
(1) New Taipei City	73	85.9 ± 9.1	0.004 * (1) > (2) (6) > (5)	65.7 ± 6.9	0.130	58.9 ± 21.4	0.393	54.2 ± 22.2	0.758	73.2 ± 14.8	0.345	72.9 ± 10.3	0.002 * (1) > (2) (1) > (4)
(2) Taichung City	31	78.3 ± 13.6		62.2 ± 5.4		50.8 ± 25.7		48.7 ± 21.7		69.1 ± 14.5		65.2 ± 11.5	
(3) Kaohsiung City	21	83.0 ± 11.7		65.0 ± 5.5		59.1 ± 21.2		52.0 ± 22.0		77.3 ± 12.0		69.4 ± 12.1	
(4) Hsinchu City	13	79.2 ± 10.3		64.1 ± 3.8		43.9 ± 17.1		43.4 ± 19.2		67.5 ± 8.1		61.5 ± 11.9	
(5) Tainan City	12	75.0 ± 13.6		63.0 ± 4.7		61.1 ± 24.1		56.5 ± 21.4		78.2 ± 12.4		64.2 ± 8.5	
(6) Taipei City	11	90.0 ± 9.7		68.5 ± 8.8		57.9 ± 22.6		47.0 ± 25.3		71.2 ± 14.9		73.1 ± 16.3	
(7) Taoyuan City	8	83.3 ± 9.7		64.0 ± 6.5		50.0 ± 31.9		46.4 ± 31.1		73.3 ± 9.9		72.5 ± 6.4	
(8) Hsinchu County	6	85.0 ± 4.0		66.3 ± 7.5		54.1 ± 12.3		52.9 ± 12.8		71.5 ± 7.9		76.6 ± 10.0	
(9) Others	12	83.0 ± 13.5		62.8 ± 8.4		56.2 ± 19.5		48.8 ± 19.6		71.9 ± 10.1		68.8 ± 8.3	
Financial status													
(1) Below the poverty line	9	78.5 ± 16.9	0.183	65.0 ± 10.3	0.709	66.6 ± 28.3	0.101	67.4 ± 25.2	0.028 * (1) > (3)	81.2 ± 13.8	0.031 * (1) > (3)	71.1 ± 14.0	0.698
(2) Middle class	151	83.8 ± 10.8		64.9 ± 6.0		56.5 ± 21.7		51.49 ± 21.0		73.0 ± 12.6		69.4 ± 11.8	
(3) Upper class	27	80.6 ± 11.0		63.7 ± 8.0		49.2 ± 23.1		44.9 ± 24.2		68.0 ± 16.0		71.2 ± 8.7	
Religion													
No religion	101	82.4 ± 11.5	0.643	64.5 ± 7.1	0.304	54.6 ± 21.5	0.065	50.4 ± 22.2	0.072	71.4 ± 13.9	0.054	70.1 ± 10.3	0.146
Buddhist	25	83.0 ± 10.5		64.0 ± 4.8		49.8 ± 18.1		45.0 ± 18.4		68.7 ± 9.0		73.9 ± 13.5	
Taoism	48	84.3 ± 11.7		64.9 ± 6.4		61.6 ± 23.8		56.5 ± 20.9		77.0 ± 13.8		67.8 ± 12.2	
Christian	8	80.8 ± 8.8		64.5 ± 2.7		47.9 ± 29.4		41.9 ± 28.1		71.2 ± 10.1		68.3 ± 8.1	
Others	5	88.6 ± 9.3		70.9 ± 4.6		71.6 ± 20.7		65.7 ± 23.2		78.6 ± 13.2		62.6 ± 16.9	
Smoking habit													
Non-smoker	156	83.0 ± 11.3	0.608	64.6 ± 6.6	0.774	55.8 ± 22.5	0.480	51.6 ± 22.0	0.217	72.2 ± 13.6	0.394	69.6 ± 11.2	0.505
Less than 1 pack per week	14	82.3 ± 11.2		64.1 ± 6.1		54.7 ± 21.4		49.7 ± 21.9		75.6 ± 12.9		68.4 ± 16.7	
1–2 packs per week	12	86.3 ± 10.7		66.3 ± 6.8		52.7 ± 22.0		42.8 ± 20.9		77.4 ± 11.8		74.0 ± 8.2	
More than 1 pack per day	5	78.6 ± 13.2		66.1 ± 8.1		70.8 ± 21.2		67.1 ± 20.4		68.0 ± 7.8		66.2 ± 5.7	
Alcohol consumption habit													
No drinking habit	63	84.0 ± 11.6	0.251	62.8 ± 6.8	0.022 *	52.5 ± 24.3	0.319	47.9 ± 24.2	0.320	69.6 ± 14.0	0.039 *	70.6 ± 10.6	0.503
Drink occasionally	113	83.0 ± 10.6		65.6 ± 6.2		57.8 ± 21.2		53.1 ± 20.9		74.7 ± 13.1		69.0 ± 12.1	
Drink more than 3 times per week	11	77.8 ± 15.0		65.9 ± 6.8		56.0 ± 21.4		51.2 ± 18.2		69.6 ± 8.2		72.3 ± 9.2	
Exercise habit													
(1) No exercise habit	36	82.5 ± 12.8	0.762	65.0 ± 7.0	0.200	53.4 ± 21.1	0.001 * (2) > (3)	48.9 ± 22.7	0.001 * (2) > (3)	70.1 ± 12.9	0.005 * (2) > (3)	70.0 ± 10.3	0.703
(2) 1–2 times per week	100	83.6 ± 11.1		65.3 ± 6.9		61.2 ± 21.7		56.5 ± 20.2		75.6 ± 13.3		69.1 ± 12.5	
(3) More than 3 times per week	51	51 ± 82.3		63.3 ± 5.3		47.3 ± 21.9		42.7 ± 22.2		68.7 ± 12.7		70.8 ± 10.1	

* *p* < 0.05.

**Table 4:** Multiple linear regression results for personal and work-related burnout.

Dependent Variable	Personal Burnout				
Independent Variable	Coefficient		Standardized Coefficient	T	*p* Value
	B	SE	β		
Constant	−32.35	12.39			
Work experience	−0.42	0.19	−0.13	−2.15	0.03
Workload	0.19	0.07	0.09	2.68	0.008
Work-related burnout	0.83	0.04	0.82	17.30	<0.001
**Dependent Variable**	**Work-Related Burnout**				
**Independent Variable**	**Coefficient**		**Standardized Coefficient**	**T**	***p* Value**
	**B**	**SE**	**β**		
Constant	10.36	12.12			
Perceived pressure	0.25	0.07	0.15	3.43	0.001
Personal burnout	0.77	0.04	0.78	17.30	<0.001

## Data Availability

The datasets will be available on reasonable request from the first author.
